# *MTO1* Worked as a Modifier in the Aminoglycosides Sensitivity of Yeast Carrying a Mitochondrial 15S rRNA C1477G Mutation

**DOI:** 10.1371/journal.pone.0124200

**Published:** 2015-04-21

**Authors:** Xiaoyu Zhu, Xiangyu He, Wei Wang, Qiyin Zhou, Zhe Yu, Yu Dai, Xufen Zhu, Qingfeng Yan

**Affiliations:** College of Life Science, Zhejiang University, Hangzhou, Zhejiang, 310058, China; Ben-Gurion University of the Negev, ISRAEL

## Abstract

*MTO1*, together with *MSS1* and *MTO2*, is a gene involved in the pathway of encoding a mitochondria-specific RNA-modifying enzyme related to the post-transcriptional modification of mitochondrial tRNAs. We have previously shown that a mutation of the *MTO2* or *MSS1* gene can suppress the neomycin-sensitive phenotype of yeast carrying a mitochondrial 15S rRNA C1477G mutation. Here we report that a null mutation of *MTO1* also can inhibit the aminoglycoside-sensitivity of yeast carrying mitochondrial 15S rRNA C1477G mutation. The C1477G mutation corresponds to the human 12S rRNA A1555G mutation. Yeast with an mtDNA C1477G mutation exhibits hypersensitivity to neomycin and displays mitochondrial function impairment beyond neomycin treatment. When the *mto1* null mutation and mitochondrial C1477G mutation coexist, the yeast strain shows growth recovery. The deletion of the nuclear gene *MTO1* regulates neomycin sensitivity in yeast carrying the mitochondrial 15S rRNA C1477G mutation. *MTO1* deletion causes the expression levels of the key glycolytic genes *HXK2*, *PFK1* and *PYK1* to become significantly up-regulated. The energy deficit due to impaired mitochondrial function was partially compensated by the energy generated by glycolysis. Being in the same pathway, the regulation of *MTO1*, *MSS1* and *MTO2* to the neomycin-sensitivity of yeast showed difference in the growth activity of strains, mitochondrial function and the expression level of glycolytic genes.

## Introduction

In both yeast and humans, the *MTO1* gene encodes an evolutionarily conserved protein which works together with the proteins encoded by the *MSS1* and *MTO2* genes to catalyze the biosynthesis of 5-carboxymethylaminomethylation (mnm^5^s^2^U34) of the wobble uridine base in mt-tRNA^Gln^, mt-tRNA^Glu^, and mt-tRNA^Lys^ [1,2]. This modification is important to the accuracy and efficiency of mtDNA translation [2]. The mitochondrial 15S rRNA C1477G mutation corresponds to the human deafness-associated mitochondrial 12S rRNA A1555G mutation [3–5]. In humans, the hearing loss phenotype is related to nuclear modifier genes such as *MTO1*, *MTO2* and *MSS1*, mtDNA mutations and to externally induced factors such as the use of Aminoglycoside antibiotics [6]. The mutation of nuclear modifier genes or the presence of Aminoglycoside antibiotics can work together with mtDNA mutation to affect the function of mitochondria. This has particular impact upon cells requiring lots of energy. However, the penetrance of hearing loss in individuals carrying the mitochondrial 12S rRNA A1555G mutation is variable, even after the treatment of Aminoglycosides [7].

Both the interaction of nuclear modifier genes with mtDNA mutations and the interaction of Aminoglycoside antibiotics with mtDNA mutations have been previously studied. However, the interaction of these three factors together remains poorly understood. In our previous study, we found that the deletion of the *MTO2* gene could significantly suppress the aminoglycoside-sensitivity of the mitochondrial 15S rRNA C1477G mutation in *Saccharomyces cerevisiae* through the up-regulation of the glycolytic pathway [8]. The *mss1* mutation have impact on the *HAP5* gene and up-regulate the expression of glycolytic transcription factors *RAP1*, *GCR1*, and *GCR2* genes to affect the sensitivity of yeast to neomycin [9]. In the pathway catalyzing the formation of the hypermodified base 5-mthyl-aminomethyl-2-thio-uridine (mnm^5^s^2^U34) in the wobble position of tRNA, *MTO2* is responsible for 2-thiolation of the U34 nucleotide [2,11], while *MTO1* and *MSS1* are responsible for the C5 substituent of the modified uridine [10]. The interactions between *MTO1*, the mitochondrial 15S rRNA C1477G mutation and Aminoglycosides is still unclear.

The yeast carrying *mto1* null mutation expressed a respiratory deficient phenotype when coexisting with the mitochondrial 15S rRNA C1477G mutation [12]. The C1477G mutation is located at the decoding site (site A) of the ribosome where codon-anticodon recognition occurs. Thus, the interaction between processed *MTO1*-dependent mt-tRNAs and ribosomal site A may be impaired by mitochondrial 15S rRNA C1477G mutation and translation could be further affected when coexisting a *mto1* null mutation [13]. In addition, the highly conserved A site is also the binding site of aminoglycoside antibiotics [14]. Aminoglycoside antibiotics inhibit ribosomal translocation where the peptidyl-tRNA moves from the A-site to the P-site, thus affecting the elongation of the polypeptide chain in translation. Human mtDNA 12S rRNA A1555G mutation and Aminoglycoside antibiotics are well known as the determinant of non-syndromic deafness [15].

To better understand the role of *MTO1*, we put the mtDNA 15S rRNA C1477G mutation, *mto1* null mutation and neomycin together as a system to find the impact of their interaction on the expression of phenotype. In our observation, the growth of *MTO1*(P^R^), carrying mitochondrial 15S rRNA C1477G mutation was significantly inhibited by the treatment of neomycin. In the yeast strain *mto1*(P^R^), which coexisting the *mto1* null mutation and mitochondrial 15S rRNA C1477G mutation, the mitochondrial function was affected dramatically. After the treatment of neomycin, *mto1*(P^R^) showed a much better growth activity than *MTO1*(P^R^). The deletion of the nuclear gene *MTO1* seemed to regulate neomycin sensitivity in yeast carrying the mitochondrial 15S rRNA C1477G mutation. In further study, the expression level of key genes in glycolytic pathway of *mto1*(P^R^) were up-regulated. The energy generated from glycolysis may compensate for the deficiencies in mitochondrial function and result in a phenotype less sensitive to neomycin.

## Materials and Methods

### Yeast strains and culture condition

The original strains were of W303-1B strain (α, *ade2*-1, *his3*-1,15, *leu2*-3,*112*, *trp1*-1, *ura3*-1) as *MTO1*(P^S^) (11) and M12-54 strain (a, *ilv5*, *trp2* [ρ^+^, P^R^_454_]) as *MYO1*(P^R^) (10). The *mto1*(P^S^) strain (α, *ade2*-1, *his3*-1,15, *leu2*-3,*112*, *trp1*-1, *ura3*-1, *mto1::HIS3*) was generated from the *MTO1*(P^S^) by the one-step gene disruption technique as described [12]. The *mto1*(P^R^) strain (α, *ade2*-1, *his3*-1,15, *leu2*-3,*112*, *mto1::HIS3* [P^R^_454_]) was generated by crossing *MTO1*(P^R^) strain with *mto1*(P^S^) ρ° strain and sporulating diploids in this study.

All the strains were cultured in YPD medium consisting of 1% yeast extract, 1% peptone and 2% glucose. Neomycin was prepared as 100mg/mL stock and then sterilized with a 0.22μm microfiltration membrane. The final working concentration of neomycin in the YPD medium was determined according to the data from a minimal inhibitory concentration assay.

### Minimal Inhibitory Concentration (MIC) Assay

The MIC assay was determined by the serial dilution method in liquid media. Yeast strains were cultured in YPD overnight at 30°C. Then the cells were diluted to a starting optical density of 0.01 OD_600_ and incubated in the presence of 2-fold gradient dilutions of neomycin sulfate. The MIC is defined as the minimal neomycin concentration at which the visible growth of yeast cells was inhibited after overnight incubation.

### Phenotype analysis and growth curve

Series dilutions (10^4^ to 10^2^) of yeast cells were spotted onto YPD plates with and without aminoglycosides. The plates were then incubated at 30°C for 3 days and photographs were taken. For growth curves, the yeast cells were cultured in liquid YPD medium only and YPD medium with neomycin. The growth rate of yeast strains were determined by first diluting the cells to 0.01 OD_600_, and then taking the samples to measure the optical density every 2 hours up to 20 hours.

### Mitochondrial respiratory rate test

Rates of oxygen consumption in intact cells were determined by Seahorse XF96 Extracellular Flux Analyzer, according to the manufacturer’s instructions. Yeast cells were cultured in liquid YPD medium with and without neomycin. The cells were then harvested and seeded in pre-coated Poly-D Lysine XF 96-well microplates (Sea-horse Bioscience) at 4×10^5^cells per well. The cells were kept in 30°C during the detection. The mitochondrial respiratory rate is shown as picomole oxygen per minute per cell (pmol/min/cell).

### Mitochondrial membrane potential assay

After the treatment of neomycin, yeast cells were harvested and compared with yeast cells cultured without neomycin. Cell pellets were suspended in 1 ml supernatant and incubated with Rhodamine 123 (5mg/mL) for 20min at 30°C in a shaker. The cells were centrifuged and washed with PBS for three times and then suspended in 20mL Phosphate Buffered Saline (PBS). A Carl Zeiss 710 LSM microscope was used for the capture of images which showed the mitochondrial membrane potential.

### Northern blot analysis

Total cellular RNA was obtained using TRIzol (Invitrogen) from the midlog phase yeast cultures (2.0×10^7^cells) according to the Manufacturer’s Instructions. Equal amounts (10μg) of total RNA were fractionated by electrophoresis through a 1.5% agarose formaldehyde gel, transferred onto a positively charged membrane (Amersham), and hybridized with a DIG-labeled *CYTB* RNA probe. RNA blots were then stripped and hybridized with DIG-labeled *COX1*, *COX2*, *ATP9*, 15S rRNA and 21S rRNA probes, respectively. As an internal control, RNA blots were stripped and hybridized with a DIG-labeled *ACTIN* probes. Other membranes were hybridized with a DIG-labeled *HXK2*, and then hybridized with DIG-labeled *PFK1* and *PYK1* probes after a striping. *ACTIN* probes had been used as an internal control.

### Western blot analysis

Total protein was obtained using RIPA buffer from log phase yeast cells after the incubation with and without neomycin. Equal amounts (50μg) of total protein were separated by SDS-PAGE, and then transferred onto a PVDF membrane. According to the manufacturer’s instructions, HXK and Tubulin (internal control) primary antibodies (Santa Cruz) and goat anti-rabbit (or rabbit anti-goat) secondary antibody were hybridized. After that, ECL solution (Santa Cruz) was used.

### Statistical Analysis

All experiments were repeated at least three times and the representative data were presented as means±SD. One-way analysis of variance (ANOVA) was performed to determine the significance between groups.

P<0.05 was considered as statistically significant: *(P<0.05), **(P<0.01).

## Results

### MIC analysis

**Fig 1:**
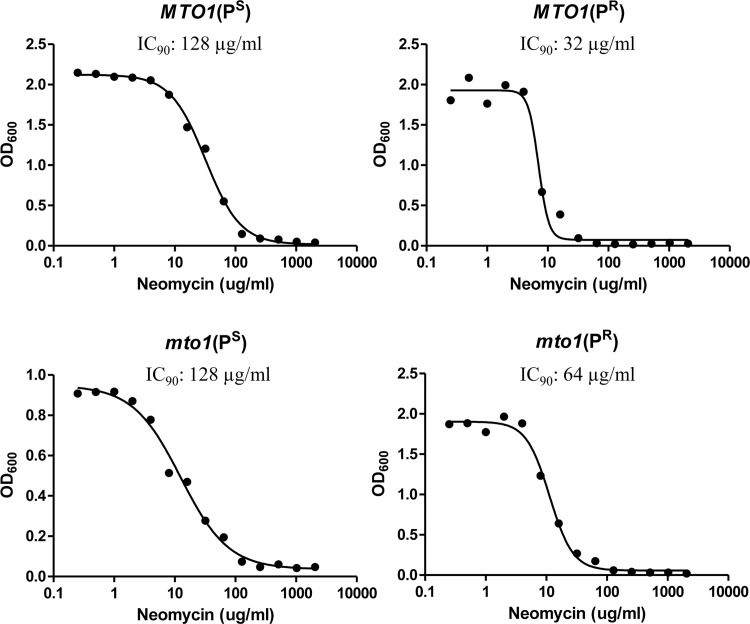
MIC analysis. The IC_90_ was determined by the serial dilution method in liquid media. The IC_90_ is defined as the neomycin concentration at which the growth of yeast cells is inhibited by 90%.

Four yeast strains with different genetic backgrounds were used for MIC analysis. The assay was determined by the serial dilution method in liquid media. Four strains showed different IC_90_ values: *MTO1* (P^S^) 128μg/mL, *mto1* (P^S^) 64μg/mL, *MTO1* (P^R^) 32μg/mL, *mto1* (P^R^) 64μg/mL (Fig 1). These showed that the four strains had different sensitivities to neomycin.

It’s known that the change of the intake of neomycin and the expression of neomycin resistance genes, such as *NEO1*, could affect the neomycin resistance of yeasts. Our results showed that the intake of neomycin of four strains had no significant difference (S1 Fig). Northern-blot assay also showed that four yeast strains had similar expression level of *NEO1* before and after the treatment of neomycin (S2 Fig). These results suggested that the neomycin intake and neomycin resistance gene expression would not be the reason of different sensitivity to neomycin in these four strains.

### Phenotype of yeast strains with/without neomycin

To understand the interaction of the nuclear modifier gene *MTO1*, the mitochondrial rRNA mutation and aminoglycoside antibiotics, yeast strains carrying mitochondrial 15S rRNA C1477G mutation and/or nuclear *mto1* null mutation were cultured in YPD medium and YPD medium containing neomycin. Both spot assay and growth curves were used for this comparison.

**Fig 2:**
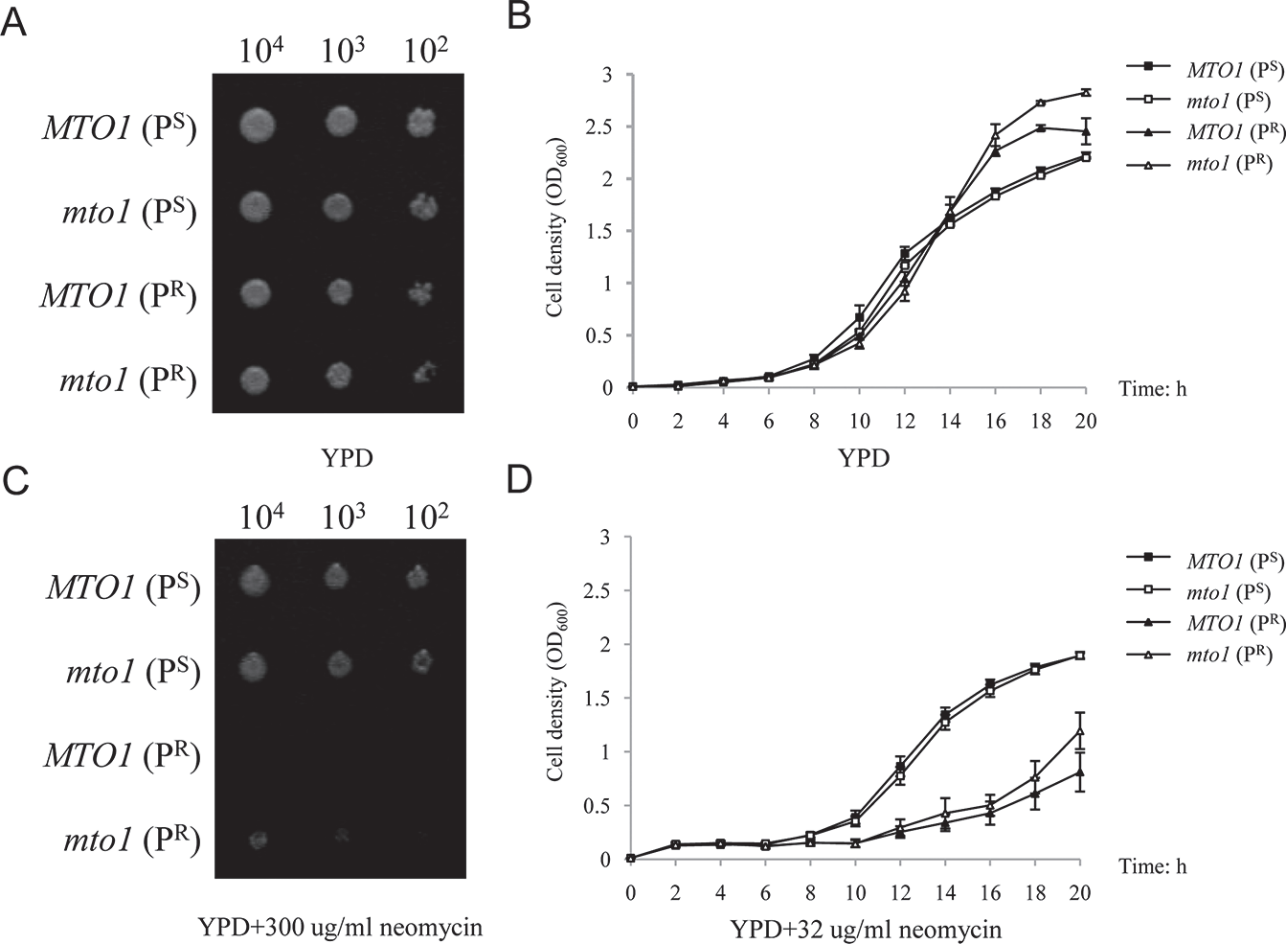
Phenotypes of different yeast strains. (A) Spot assay of four yeast strains. The assay was performed by spotting decreasing concentrations of yeast cells (10^4^,10^3^, and 10^2^) on a 2% glucose medium (YPD). (B) Growth curves of yeast strains in YPD for 20 hours. (C) Spot assay of four yeast strains on YPD medium containing 300μg/mL neomycin. (D) Growth curves of yeast strains on YPD medium containing 32μg/mL neomycin.

From the results, all four strains with different genetic backgrounds showed normal growth on YPD medium (Fig 2A). After adding 300μg/mL neomycin in the YPD medium, the growth of the strain *MTO1* (P^R^), which carrying mitochondrial 15S rRNA C1477G mutation, was affected significantly (100% inhibition). However, the strain *mto1*(P^R^), which carrying both *mto1* null mutation and mitochondrial 15S rRNA C1477G mutation, showed a much better growth activity (Fig 2C). The P^S^ strains, whose mitochondrial 15S rRNA had no mutation, showed no susceptibility to the treatment of neomycin. There was no significant difference between *MTO1*(P^S^) and *mto1*(P^S^) in spite them having a different nuclear background.

The results of the yeast growth curve in liquid media confirmed this result by spot assay (Fig 2B and Fig 2D). These four strains showed similar growth curves during culture in a YPD medium. While in YPD medium containing 32μg/mL neomycin, the P^S^ strains entered into the logarithmic phase after 6 hours’ incubation. The P^R^ strains entered into the logarithmic phase after 10 hours’ incubation. The growth speed of *mto1*(P^R^) was much better than that of *MTO1*(P^R^). These data indicates that the deletion of nuclear gene *MTO1* regulates neomycin sensitivity in yeast carrying the mitochondrial 15S rRNA C1477G mutation.

### Mitochondrial function shown by mitochondrial respiratory rates

**Fig 3:**
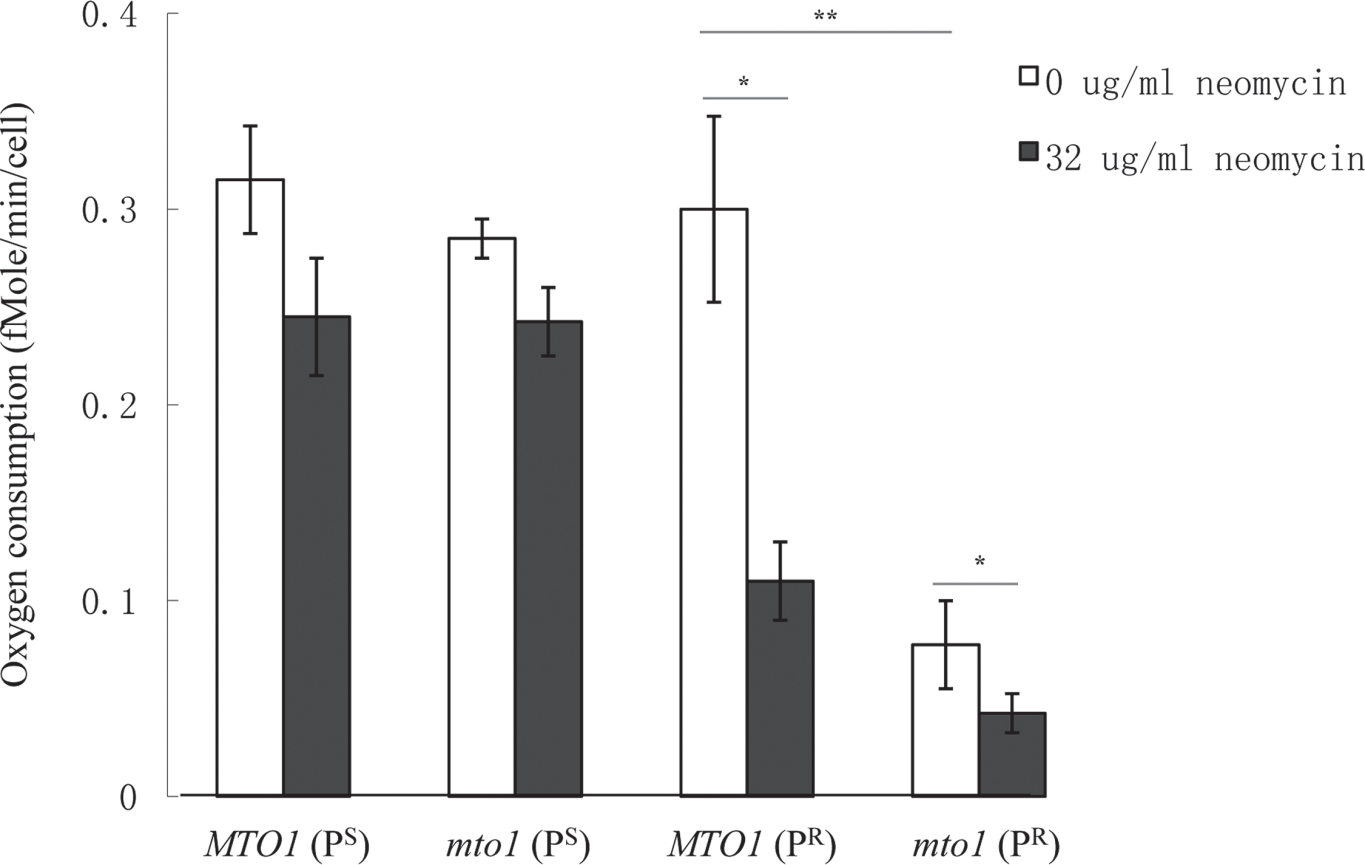
Mitochondrial respiratory rate of yeast strains. Yeasts cultured in the medium with or without 32μg/ml neomycin were harvested in the log-phase. The oxygen consumption rate of each yeast strain was measured using a FX-96 oxygraph (SeaHorse Biosciences). The cell density was 4×10^5^cells per well. Values are the mean of three independent experiments. Data are represented as mean ± SD. *: p<0.05. **: p<0.01.

The mitochondrial respiratory rate is usually a symbol of mitochondrial function. The respiratory rates of the four strains were measured by detecting the rate of oxygen consumption with Seahorse XF96 Extracellular Flux Analyzer (Fig 3). In YPD medium, the respiratory rate of wild-type strain *MTO1*(P^S^) was 0.315fmol/min/cell. A light impact on mitochondrial function was shown in both the strains carrying the *mto1* null mutation and in those carrying the mitochondrial 15S rRNA C1477G mutation. The respective respiratory rates of *mto1*(P^S^) and *MTO1*(P^R^) were similar to that of *MTO1*(P^S^). When the *mto1* null mutation and mitochondrial 15S rRNA C1477G mutation coexisted, the respiratory rate of *mto1*(P^R^) was affected dramatically to a level of 75% lower than the respiratory rate of *MTO1*(P^S^).

After the treatment with 32μg/mL neomycin, the respiratory rate of *MTO1*(P^S^) and *mto1*(P^S^) had declined by 22% and 15% respectively. Neomycin had more of an impact on the mitochondrial function of the P^R^ strains whose mitochondrial 15S rRNA had the C1477G mutation. The respiratory rate of *MTO1*(P^R^) and *mto1*(P^R^) decreased by 63% and 45% respectively after the treatment of neomycin. This result suggested that yeast cells carrying the mitochondrial 15S rRNA mutation were more sensitive to neomycin than yeasts with wild type mitochondria.

### Mitochondrial Membrane Potential

The mitochondrial membrane potential is critical for maintaining the physiological function of the respiratory chain to generate ATP. Thus, the detection of mitochondrial membrane potential can provide important clues about the physiological status of the cell and the function of the mitochondria. In this Rhodamine 123 fluorescent dye can be used as an indicator.

**Fig 4:**
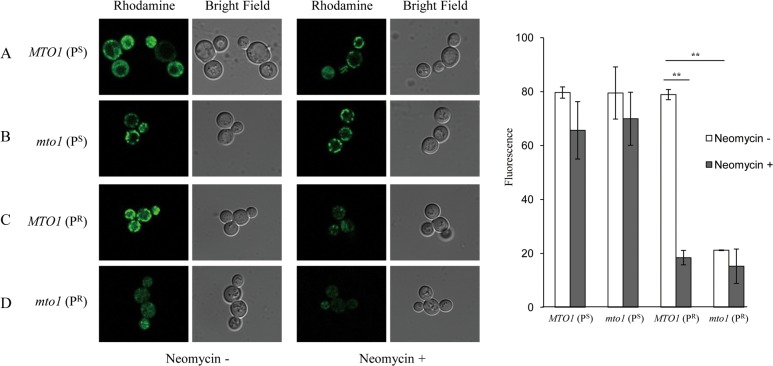
Mitochondrial membrane potential, shown by Rhodamine 123 staining. Yeasts cultured in the medium with or without 32μg/mL neomycin were harvested in the log-phase. The cells were incubated with Rhodamine 123 (5μg/mL) for 20min at 30°C, and then harvested. The cell pellets were suspended in 20mL PBS and visualized using Carl Zeiss 710 LSM microscopy. The fluorescence signal is shown to the far right of the graph. Data are represented as mean ± SD. **: p<0.01.

In the YPD medium, *MTO1*(P^S^), *mto1*(P^S^) and *MTO1*(P^R^) showed a strong fluorescent signal. This indicates that they had a high mitochondrial membrane potential (Fig 4). The fluorescent signal of *mto1*(P^R^) was much lower, being only 27% of *MTO1*(P^S^). This is an indication that the mutations, both in *mto1* gene and mitochondrial 15S rRNA, significantly affect the function of mitochondria.

After culturing in the YPD medium containing 32μg/mL neomycin, the fluorescent signal of *MTO1*(P^S^) and *mto1*(P^S^) slightly decreased. By contrast, the fluorescent signal of *MTO1*(P^R^) significantly decreased by 77% when compared to the cells without treatment of neomycin. The mitochondrial membrane potential of *mto1*(P^R^) was much lower in the cells without treatment of neomycin than the other three strains, and the fluorescent signal further decreased after the treatment of neomycin. This change is visible and suggests that the mitochondrial function of yeasts carrying the mitochondrial 15S rRNA mutation could be significantly affected by neomycin. After the treatment of neomycin, the mitochondrial function of *mto1*(P^R^) was the weakest in the comparison set. This result is consistent with the results of the mitochondrial respiratory rate test.

### Transcription level of mitochondrial genes

**Fig 5:**
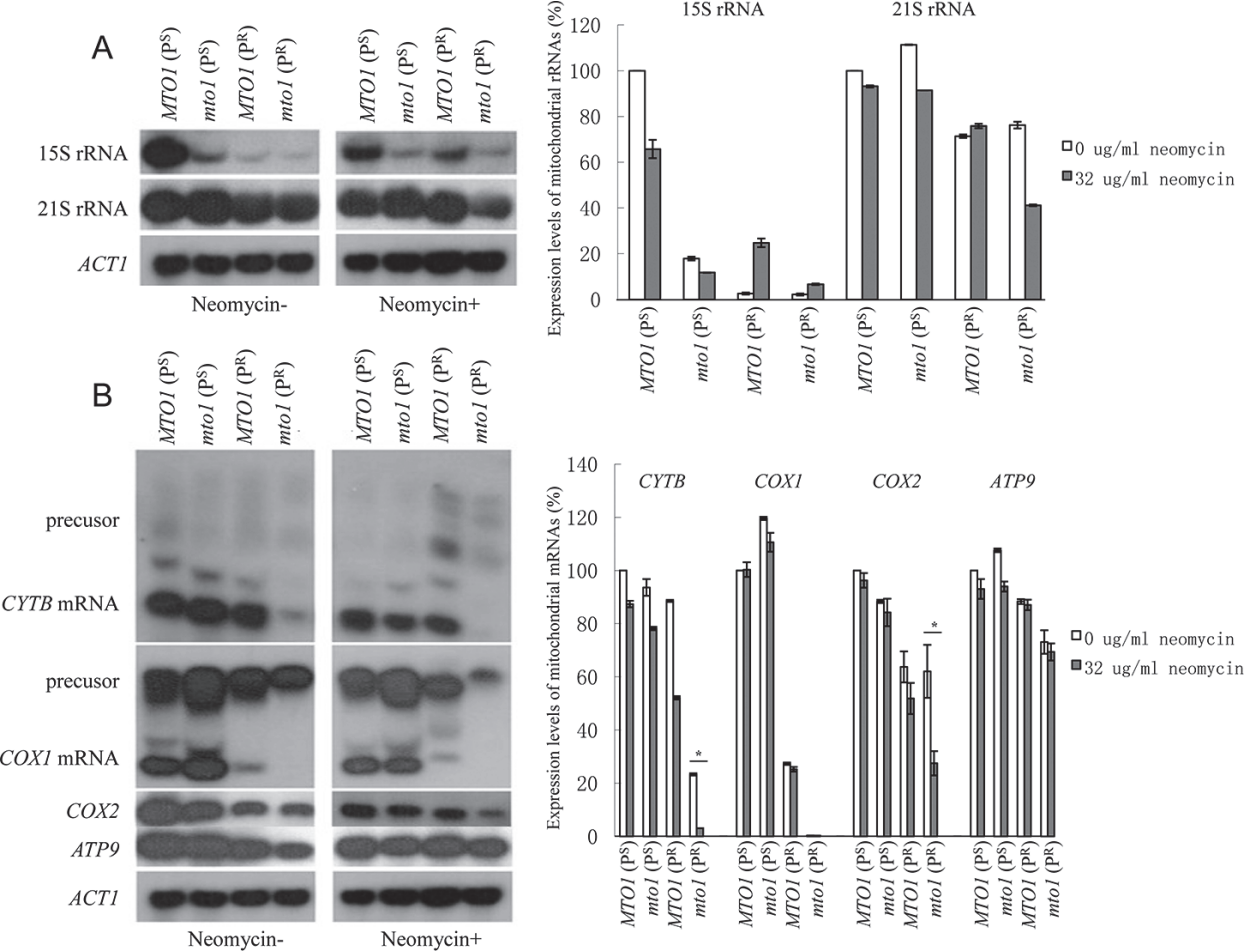
Transcription level of mitochondrial genes. (A) Northern blot analysis of mitochondrial 15S rRNA and 21S rRNA, *ACT1* as the internal control. Quantitative analysis is shown in the graphs to the right. (B) Northern blot analysis of *CYTB*, *COX1*, *COX2*, *ATP9*, *ACT1* as the internal control. Quantitative analysis is shown in the right graph. Data are represented as mean ± SD. *: p<0.05.

Northern blot was used to show the impacts of the *mto1* null mutation, mitochondrial 15S rRNA C1477G mutation and neomycin on the transcription level of mitochondrial genes. In the absence of neomycin, the transcription levels of 15S rRNA in *MTO1*(P^R^) and *mto1*(P^R^) were dramatically lower than those of the *MTO1*(P^S^) strain (Fig 5A). The C1477G mutation may impair the stability of 15S rRNA. However, the 15S rRNA transcript of *mto1*(P^S^) was also lower than *MTO1*(P^S^). This suggests that the *mto1* null mutation may have an impact on 15S rRNA. The transcription of 21S rRNA showed no significant difference between the 4 strains without neomycin. In the presence of neomycin, the transcription level of 15S rRNA and 21S rRNA in *MTO1*(P^S^) and *mto1*(P^S^) slightly decreased. *mto1*(P^R^) showed inhibited transcription of 21S rRNA, however, neomycin didn’t show inhibitory effect on 15S rRNA in both of *MTO1*(P^R^) and *mto1*(P^R^) strains.

While in the genes *CYTB*, *COX1*, *COX2* and *ATP9*, the four strains showed different expression levels (Fig 5B). Two P^S^ strains showed strong transcription levels in these four genes. The transcription of these genes slightly decreased upon the treatment with neomycin. The P^S^ strains had normal mitochondrial function and the function was not significantly affected by neomycin. When there is a mitochondrial 15S rRNA C1477G mutation, *MTO1*(P^R^) showed a weaker expression level than the P^S^ strains, especially in *COX1* mRNA. *COX1*, as well as other genes encoded by mitochondrial DNA, contains exons and introns, so there are RNA precursors and mature mRNA could be detected. After the treatment of neomycin, the precursor of *COX1* was further reduced. The mitochondrial 15S rRNA C1477G mutation had some impact on mitochondrial function and the function could be further affected by neomycin. When the mitochondrial 15S rRNA C1477G mutation and *MTO1* null mutation coexisted, the transcription level of all four genes showed an obvious decline in *mto1*(P^R^). Only a precursor of *COX1* and very low levels of *CYTB* expression could be found. The *mto1* null mutation further affected the expression of mitochondrial genes and the maturation of *COX1* and *CYTB* primary transcripts when compared with *MTO1*(P^R^). After the treatment of neomycin, the transcription level of *CYTB* and *COX2*, and the precursor of *COX1* further decreased. The mitochondrial function was significantly affected.

### Expression level of key genes in glycolytic pathway

**Fig 6:**
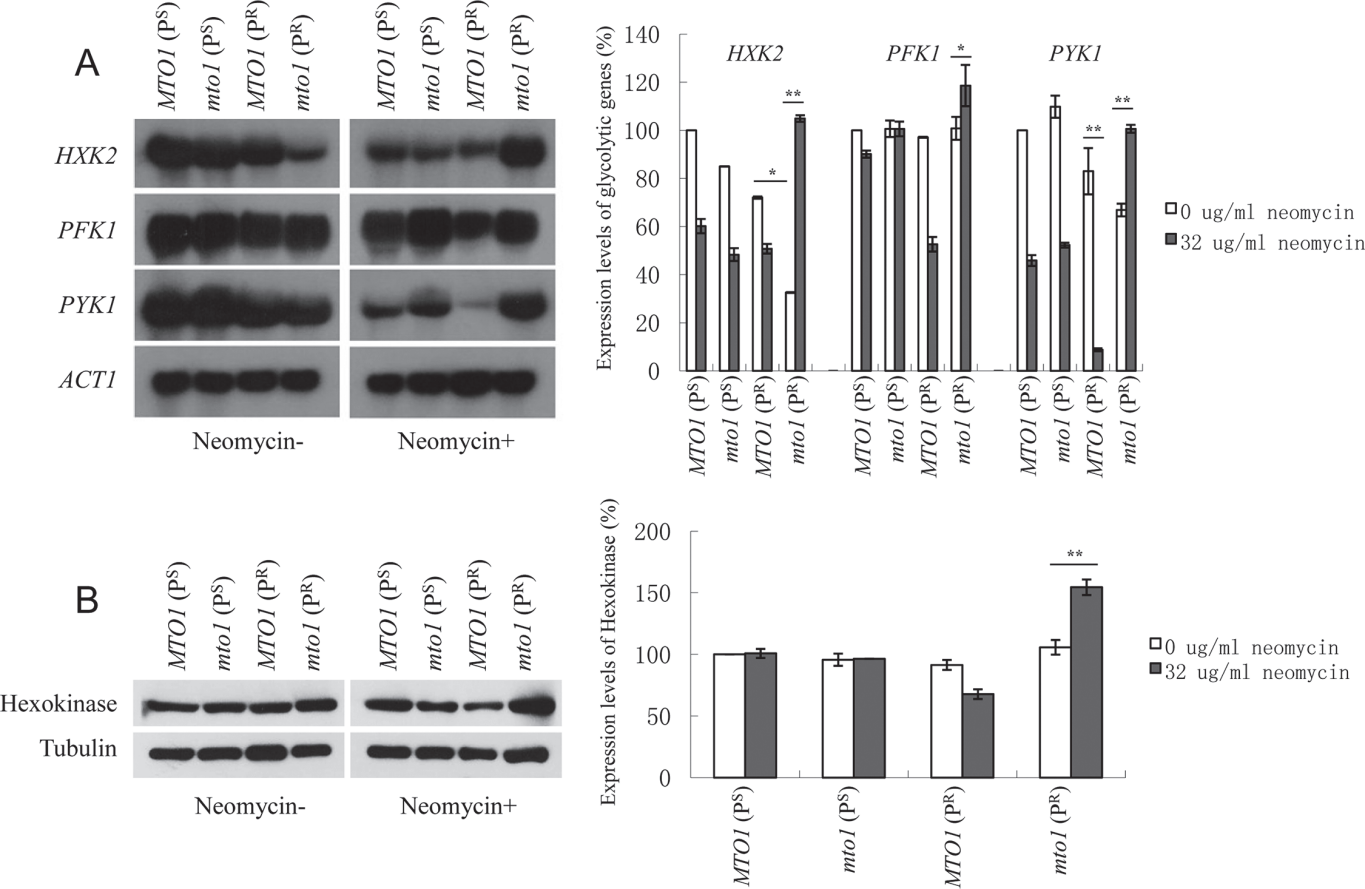
Expression level of key glycolytic genes. (A) Northern blot analysis of *HXK2*, *PFK1*, *PYK1*, with *ACT1* as the internal control. Quantitative analysis is shown in the graph to the right. (B) Western blot analysis of Hexokinase, Tubulin as the internal control. Quantitative analysis is shown in the right graph. Data are represented as mean ± SD. *: p<0.05. **: p<0.01.

Glycolysis is an important pathway which is related to ATP generation. In the glycolytic pathway, the three regulated enzymes are hexokinase, phosphofructokinase, and pyruvate kinase. Northern blot was used to detect the expression level of these three enzymes with *Actin* as the internal control (Fig 6A).

Without neomycin, the four strains showed similar expression levels of *PFK1* and *PYK1*. The *mto1*(P^R^) strain showed a weaker expression level of *HXK2* when compared to the other three strains. After the treatment with neomycin, the expression of *mto1*(P^R^) in *HXK2* had dramatically increased by 225%. The other three strains showed no significant difference. In *PFK1* and *PYK1*, the expression of *mto1*(P^R^) increased by 17% and 34% but the expression level of *MTO1*(P^R^) decreased.

To confirm this result, western blot was used to analyze the expression level of hexokinase, the key enzyme in the glycolytic pathway (Fig 6B). When cultured in media without neomycin, the hexokinase expression of four strains showed similar levels. After neomycin treatment, it showed no impact on *MTO1*(P^S^) and *mto1*(P^S^) strains. However, the hexokinase expression of *MTO1*(P^R^) showed a decline. Only in *mto1* (P^R^), did the hexokinase expression show a significant increase where it rose by 46%.

To summarize, the *MTO1*(P^R^) strain with the mitochondrial 15S rRNA C1477G mutation is sensitive to the effect of neomycin. Its mitochondrial function is significantly inhibited by the treatment of neomycin. In the *mto1*(P^R^), the strain having both the mitochondrial 15S rRNA C1477G mutation and *mto1* null mutation, glycolysis is up-regulated. This effect may be a compensation for weak mitochondrial function leading to the observation that this phenotype is less sensitive to neomycin.

## Discussion

### *mto1* mutation activates the glycolytic pathway to affect the neomycin sensitivity of yeast carrying the mitochondrial 15S rRNA C1477G mutation

The mitochondrial 15S rRNA C1477G mutation is well-known to be related to a mitochondrial dysfunction when coexisting with the null mutation of *MTO1*, *MTO2* or *MSS1* [5,18–20]. The C1477G mutation locates at the decoding site (site A) of the ribosome where the codon-anticodon recognition occurs [21]. The precise codon-anticodon pairing that occurs during translation also requires a post-transcriptional modification at the wobble position of the tRNA [22,23]. This modification is known to be related to *MTO1*. Our results show a significantly decreased mitochondrial respiratory rate and mitochondrial membrane potential. This suggests the mitochondrial function is significantly affected by the 15S rRNA C1477G mutation and *mto1* mutation.

Our study also shows that yeast cells carrying the mitochondrial 15S rRNA C1477G mutation exhibit hypersensitivity to neomycin, one member of the Aminoglycosides. As a comparison, the growth and the mitochondrial function of P^S^ cells were not significantly affected by the treatment of neomycin. The mutation may change the structure of this position and have further impact on the interaction with neomycin, whose binding site is in site A [24,25].

However, the effect of neomycin and the *MTO1* mutation did not work together with a 15S rRNA C1477G mutation to further inhibit the viability of yeast cells. The *mto1*(P^R^) strain showed less sensitivity to neomycin than the *MTO1*(P^R^) strain. We observed that the transcription levels of *HXK2*, *PFK1* and *PYK1* in *mto1*(P^R^) strains were more highly up-regulated than *MTO1*(P^R^) strains. In this strain, the deletion of the *MTO1* gene may activate the glycolytic metabolic pathway so that energy generated by the glycolysis pathway may compensate and lessen the energy deficit due to impaired oxidative phosphorylation. The sensitivity to neomycin in *mto1*(P^R^) strains are then decreased.

### Comparison of the effect of *MTO1*, *MTO2* and *MSS1* genes involved in the same pathway

Modified nucleosides are a characteristic structural feature of tRNAs. These post-transcriptional nucleoside modifications are required for the stability and proper function of tRNAs [26,27]. The precise codon-anticodon pairing in translation requires a post-transcriptional modification at the wobble position of the tRNA. *MTO1*, *MSS1* and *MTO2* genes are involved in the pathway of mitochondria-specific RNA-modifying enzymes which is related to the biosynthesis of the wobble base. In *Escherichia coli*, two enzymes, MnmE (a homolog of *MSS1*) and GidA (a homolog of *MTO1*), form a protein complex for synthesizing cmnm5U [27–29]. In yeast, *MTO1* and *MSS1* have been reported to be responsible for the C5 substituent of the modified uridine which is the initial step of biosynthesis [2]. In this pathway, *MTO2* was reported to be a mitochondrial-specific 2-thiouridylase that is responsible for the 2-thiolation of the wobble position in human and yeast mt tRNAs [8].

In our previous study, yeast strains having *mto2* or *mss1* null mutations together with the mitochondrial 15S rRNA C1477G mutation showed decreased sensitivity to aminoglycosides. Now we show that the *mto1* null mutation also had significant impact on the neomycin sensitivity of yeast carrying the 15S rRNA C1477G mutation. However, the phenotype of *mto2* or *mss1* strains is different from that which had been previously observed in *mto1* strains, even though these three genes occur in the same pathway. From the phenotype, the recovery of *mss1*(P^R^) compared to *MSS1*(P^R^) is significant and *mto2*(P^R^) showed a much better growth activity than *MTO2*(P^R^), and the last one is *mto1*(P^R^) compared to *MTO1*(P^R^).

When comparing the results of mitochondrial respiratory rates, a similar trend can be seen in the yeast strains of *MTO1*, *MTO2* and *MSS1*. Before the treatment of neomycin, *MTO1*(P^R^), *MTO2*(P^R^) and *MSS1*(P^R^) showed normal mitochondrial function. However, the mitochondrial respiratory rate of *mto1*(P^R^), *mto2*(P^R^) and *mss1*(P^R^) were 25%, 27% and 17% of those of *MTO1*(P^S^), *MTO2*(P^S^) and *MSS1*(P^S^), respectively. The combination of nuclear gene null mutation and mitochondrial 15S rRNA C1477G mutation significantly affected the function of mitochondria. When treated with neomycin, the mitochondrial respiratory rate of *MTO1*(P^R^), *MTO2*(P^R^) and *MSS1*(P^R^) had decreased to only 35%, 30% and 43% of *MTO1*(P^S^), *MTO2*(P^S^) and *MSS1*(P^S^), respectively. The yeast strains carrying mitochondrial 15S rRNA C1477G mutation showed a hypersensitivity to neomycin. The mitochondrial function of *mto1*(P^R^), *mto2*(P^R^) and *mss1*(P^R^) were further affected, showing only 13%, 11% and 16% mitochondrial respiratory rate of *MTO1*(P^S^), *MTO2*(P^S^) and *MSS1*(P^S^) respectively.

In the study of the glycolytic pathway, the expression level of glycolytic genes *HXK2*, *PFK1* and *PYK1* in *MTO1*(P^R^), *MTO2*(P^R^) and *MSS1*(P^R^) were decreased after the treatment of neomycin. In *mto1*(P^R^), *mto2*(P^R^) and *mss1*(P^R^) strains, the decreased sensitivity to neomycin was related to the activation of the glycolytic metabolic pathway. The energy generated by the glycolysis pathway was provided to the strains with impaired oxidative phosphorylation. However, the extent of the recovery of *mto1*(P^R^), *mto2*(P^R^) and *mss1*(P^R^) strains were not identical. This may be due to their respective and relative expression levels of glycolytic genes. In *mto1*(P^R^), if the expression level of *MTO1*(P^S^), without neomycin, was defined as 100%, the expression level of *HXK2*, *PFK1* and *PYK1* were 33%, 101% and 67% before the treatment of neomycin and increased to 105%, 119% and 101% after the treatment of neomycin, respectively. The expression of *HXK2* and *PYK1* increased significantly. In *mto2*(P^R^), taking the expression level of *MTO2*(P^S^) without neomycin as 100%, the expression level of *HXK2*, *PFK1* and *PYK1* were 88%, 69% and 110% before the treatment of neomycin and increased to 166%, 146% and 166% after the treatment of neomycin, respectively. Here, the expressions of all three genes were notably increased. However, the recovery of *mto2*(P^R^) was much better than that of *mto1*(P^R^) from this phenotype. In *mss1*(P^R^), taking the expression level of *MSS1*(P^S^) without neomycin as 100%, the expression level of *HXK2*, *PFK1* and *PYK1* were 102%, 110% and 109% before the treatment of neomycin and 72%, 157% and 98% after the treatment of neomycin. Here the expression of *PFK1* had increased. This differed from the case of *mto1*(P^R^) whose expression levels of *HXK2* and *PYK1* were increased. The recovery of *mss1*(P^R^) in the phenotype was the best. This was also believed to be related to the expression of *HAP5* which could up-regulate the expression of glycolytic transcription factor.

The expression level of Hexokinase also showed some differences in three strains. The expression level of *mto2*(P^R^) compared to *MTO2*(P^S^) was higher than *mto1*(P^R^) compared to *MTO1*(P^S^). In addition, *mss1*(P^R^) failed to show any higher expression level of Hexokinase. This result, taken from proteins, was consistent with the RNA expression level observations. These results are a sign that these three genes have different functions, despite occurring in the same pathway.

*MTO1* encodes an enzyme involved in the post-transcriptional modification of mitochondrial tRNAs. Thus, *MTO1* is related to the accuracy and efficiency of mtDNA translation. In a study of deafness, the *MTO1* gene was defined as one nuclear modifier gene which interacted with other factors, such as mitochondrial mutations, to affect the deafness phenotype. Another study has shown that *MTO1* mutations are associated with a mitochondrial disorder characterized by hypertrophic cardiomyopathy, lactic acidosis, and MRC deficiency [16]. Recently, the *MTO1* gene was also reported to play a role in breast cancer tissues and cells [17]. This indicates that the function of the *MTO1* gene may be more complicated than previously considered. Though our study also gives a new insight into the function of *MTO1*, further work is required to continue to investigate the mechanisms underlying the enzymes *MTO1* encodes or the enzymatic pathway as it relates to mitochondria.

## Supporting Information

S1 Fig Intake of neomycin. An aminoglycoside hypersensitive E.coli strain TOP10 (Invitrogen) was used to test the residual antibiotic concentration in the YPD media. The YPD medium with neomycin was collected after the treatment of four strains. The residual concentration of agent was read from an aminoglycoside standard curve (R2 = 0.99).
(TIF)

S2 Fig Expression level of NEO1. Northern blot analysis of NEO1, with ACT1 as the internal control. Quantitative analysis is shown in the graph to the right. Data are represented as mean ± SD.
(TIF)

S1 Materials and Methods (DOCX)
