# Supplementary figures and images for: MTO1 Worked as a Modifier in the Aminoglycosides Sensitivity of Yeast Carrying a Mitochondrial 15S rRNA C1477G Mutation
Source: PLoS One. 2015 Apr 21;10(4):e0124200. doi: 10.1371/journal.pone.0124200 (PMC4405582; doi:10.1371/journal.pone.0124200)

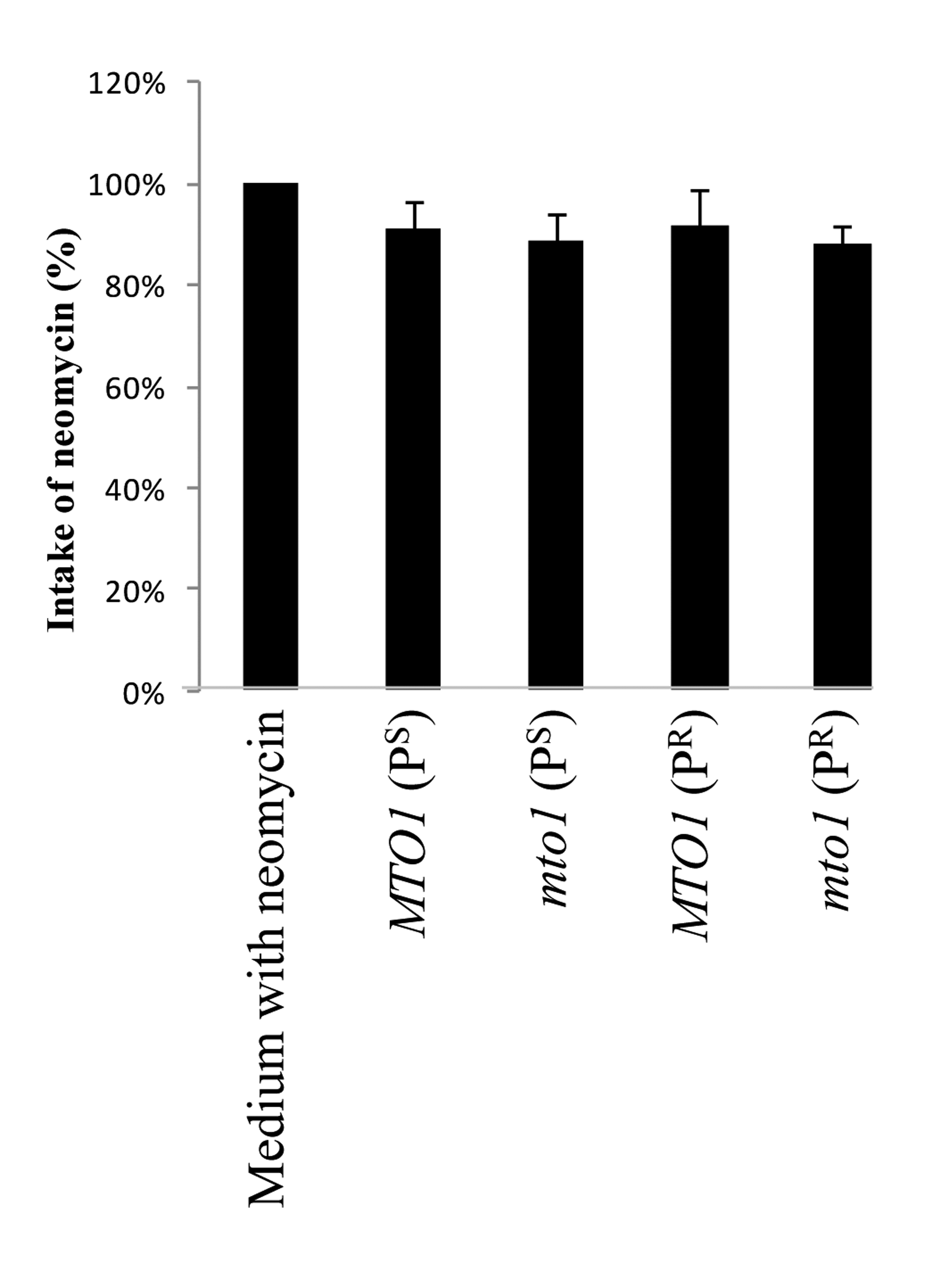

Supplement: S1 Fig — An aminoglycoside hypersensitive E.coli strain TOP10 (Invitrogen) was used to test the residual antibiotic concentration in the YPD media. The YPD medium with neomycin was collected after the treatment of four strains. The residual concentration of agent was read from an aminoglycoside standard curve (R2 = 0.99). (TIF) [file pone.0124200.s001.tif]

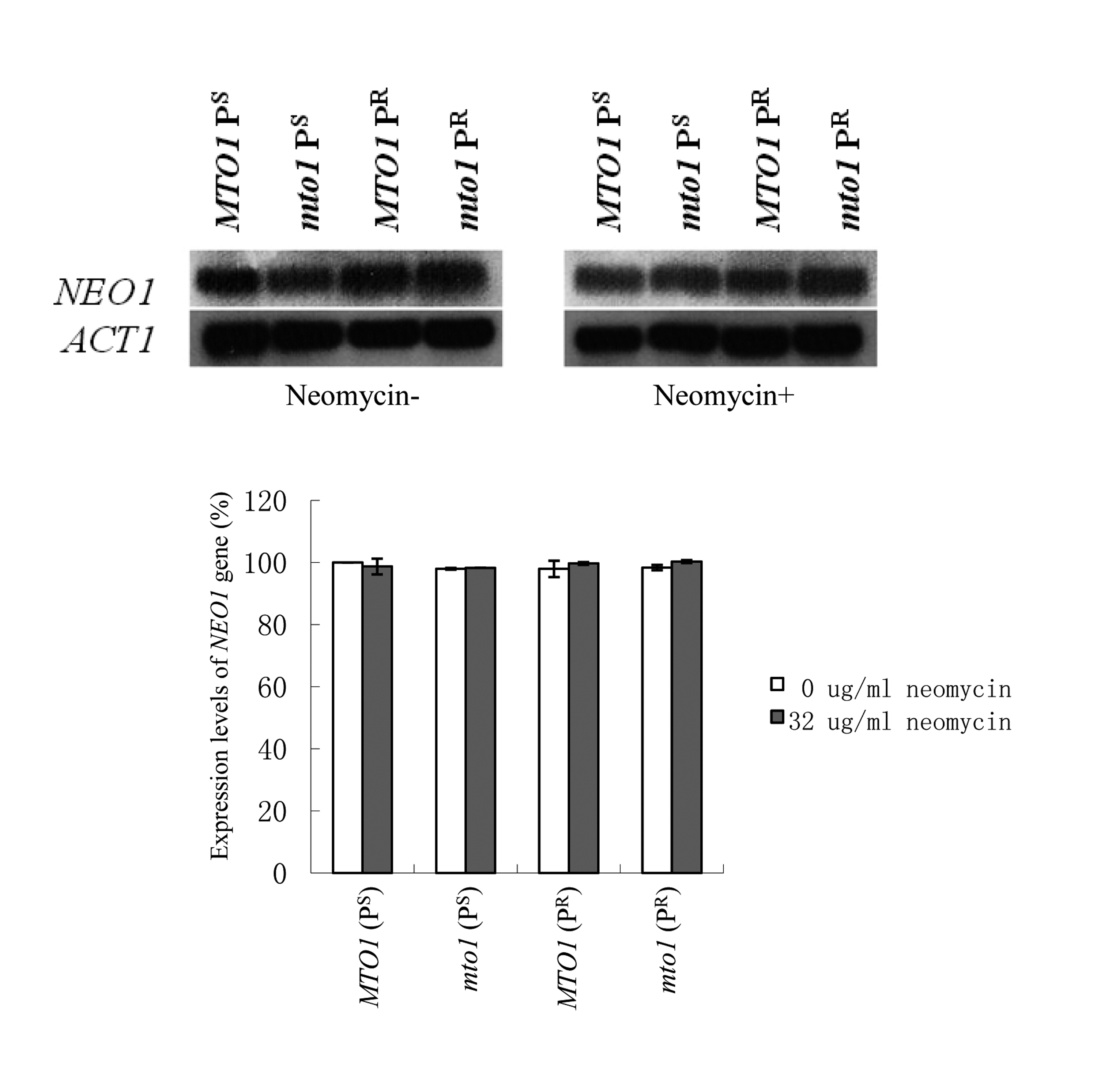

Supplement: S2 Fig — Northern blot analysis of NEO1, with ACT1 as the internal control. Quantitative analysis is shown in the graph to the right. Data are represented as mean ± SD. (TIF) [file pone.0124200.s002.tif]
