# Supplementary material for: MTO1 Worked as a Modifier in the Aminoglycosides Sensitivity of Yeast Carrying a Mitochondrial 15S rRNA C1477G Mutation
Source: PLoS One. 2015 Apr 21;10(4):e0124200. doi: 10.1371/journal.pone.0124200 (PMC4405582; doi:10.1371/journal.pone.0124200)
Supplement: S1 Materials and Methods — (DOCX) [file pone.0124200.s003.docx]

**Supplementary information**

**Materials and methods**

**Construction of yeast strains**

In this study, the original strains were W303-1B strain (α*, ade2*-1, *his3*-1,15, *leu2*-3,*112*, *trp1*-1, *ura3*-1) as *MTO1*(P^S^) [1] and M12-54 strain (a, *ilv5*, *trp2* [ρ^+^, P^R^_454_]) as *MYO1*(P^R^) [2].

*mto1*(P^S^) strain (α*, ade2*-1, *his3*-1,15, *leu2*-3,*112*, *trp1*-1, *ura3*-1, *mto1::HIS3*) was constructed from the *MTO1*(P^S^). The 1583bp fragment containing the full-length coding region of *MTO1* (NM_001181102.1) was amplified by PCR and total yeast genomic DNA isolated from *MTO1*(P^S^) cells as a template, with primers containing BamHⅠ enzyme restriction sites (5’-3’) GGATCCATGCTGCGTGTAACAACCT (nt 1-19) and GGATCCTGACTGGTTGGCTTGGCT (nt 1988-2005). The PCR product was ligated to the vector pGEM-7Zf(+) (Promega) to produce the plasmid pGEM-MTO1. A 669bp Bsg l fragment (positions 598-1267) spanning the coding region of *MTO1* was deleted by Bsg I and 1.2kb fragment containing the full-length coding region of *HIS3* was ligated into the Bsg l site of pGEM-MTO1. The resultant plasmid containing the *mto1::HIS3* allele was digested with BamHⅠ, and the fragment was introduced into wild type yeast strain *MTO1*(P^S^) using LiAc-DMSO method [3]. The integrated deletion construct *mto1*(P^S^) was selected for cells growing on glucose-minimal medium in the absence of histidine [4].

*mto1*(P^R^) strain (α, *ade2*-1, *his3*-1,15, *leu2*-3,*112*, *mto1::HIS3* [P^R^_454_]) was constructed by crossing *MTO1*(P^R^) strain with *mto1*(P^S^) ρ^o^ strain isolated with ethidium bromide to cause the loss of mtDNA (ρ^o^). The resulting diploids were sporulated and products of meiosis were dissected onto glucose medium according to the general procedures [5] and elsewhere described [1,4,6]. Meiotic progeny derived from the cross was examined for the phenotype.

**Intake of neomycin**

An aminoglycoside hypersensitive *E.coli* strain TOP10 (Invitrogen) was used to test the residual antibiotic concentration in the YPD media. The YPD medium with neomycin was collected after the treatment of four strains. The medium was harvested and the yeast cells were filtered with 0.22μm filter membrane. Steel cups containing 200μL medium were placed onto the Luria-Bertani plate on which the TOP10 strain was spread earlier. The plates were cultured at 37℃ for 15-18 hours and the diameters of each inhibition zone were measured. The residual concentration of agent was read from an aminoglycoside standard curve (R^2^=0.99).

**Expression level of *NEO1***

Total cellular RNA was obtained from the midlog phase yeast cultures (2.0×10^7^ cells) by using TRIzol (Invitrogen) according to the manufacturer’s instructions. Equal amounts (10μg) of total RNA were fractionated by electrophoresis through a 1.5% agarose formaldehyde gel, transferred onto a positively charged membrane (Amersham), and hybridized with a DIG-labeled *NEO1* RNA probe. As an internal control, RNA blots were stripped and hybridized with a DIG-labeled *ACTIN* probes.

**Results**

**Intake of neomycin**

The four strains internalized around 10% of neomycin added to the medium. Similar neomycin concentration could be found in the medium after the treatment of yeasts, which suggested that the intake of neomycin of four strains had no significant difference (S1_Fig).

**Expression level of *NEO1***

The expression level of *NEO1*, which is a gene related to neomycin resistance, was observed. Northern-blot assay showed that four yeast strains were similar expression level of *NEO1* before and after the treatment of neomycin (S2_Fig). These results suggested that these four strains had similar neomycin resistance gene expression, and it would not be the reasons of the results in this manuscript.

**Reference:**

1. Colby G, Wu M, Tzagoloff A. *MTO1* codes for a mitochondrial protein required for respiration in paromomycin-resistant mutants of *Saccharomyces cerevisiae*. J Biol Chem. 1998; 273: 27945-27952.

2. Decoster E, Vassal A, Faye G. MSS1, a nuclear-encoded mitochondrial GTPase involved in the expression of COX1 subunit of cytochrome c oxidase. J Mol Biol. 1993; 232: 79-88.

3. Gietz RD, Schiestl RH. Applications of high efficiency lithium acetate transformation of intact yeast cells using single-stranded nucleic acids as carrier. Yeast. 1991; 7: 253-263.

4. Yan Q, Li X, Faye G, Guan MX. Mutations in *MTO2* related to tRNA modification impair mitochondrial gene expression and protein synthesis in the presence of a paromomycin resistance mutation in mitochondrial 15S rRNA. J Biol Chem. 2005; 280: 29151-29157.

5. Sherman F, Fink GR, Hicks JB. Methods in Yeast Genetics, Cold Spring Harbor Laboratory, Cold Spring Harbor, NY. 1983

6. Séraphin B, Boulet A, Simon M, Faye G. Construction of a yeast strain devoid of mitochondrial introns and its use to screen nuclrar genes involved in mitochondrial splicing. Proc Natl Acad Sci USA. 1987; 84: 6810-6814.
